# P‐glycoprotein overexpression in bone marrow–derived multipotent stromal cells decreases the risk of steroid‐induced osteonecrosis in the femoral head

**DOI:** 10.1111/jcmm.12917

**Published:** 2016-07-11

**Authors:** Ning Han, Zengchun Li, Zhengdong Cai, Zuoqin Yan, Yingqi Hua, Chong Xu

**Affiliations:** ^1^ Shanghai East Hospital of Tongji UniversityShanghaiChina; ^2^ Shanghai Tenth People's Hospital of Tongji UniversityShanghaiChina; ^3^ Shanghai First People's Hospital of Jiaotong UniversityShanghaiChina; ^4^ Zhongshan Hospital of Fudan UniversityShanghaiChina

**Keywords:** P‐glycoprotein, bone marrow–derived multipotent stromal cell, adipogenesis, osteogenesis, osteonecrosis

## Abstract

P‐glycoprotein (P‐gp) plays a role in steroid‐induced osteonecrosis of the femoral head (ONFH), but the underlying mechanism remains unknown. We hypothesized that P‐gp overexpression can prevent ONFH by regulating bone marrow–derived multipotent stromal cell (BMSC) adipogenesis and osteogenesis. BMSCs from Sprague–Dawley rats were transfected with green fluorescent protein (GFP) or the multidrug resistance gene 1 (MDR1) encoding GFP and P‐gp. Dexamethasone was used to induce BMSC differentiation. Adipogenesis was determined by measuring peroxisome proliferator‐activated receptor (PPAR‐γ) expression and the triglyceride level. Osteogenesis was determined by measuring runt‐related transcription factor 2 (Runx2) expression and alkaline phosphatase activity. For *in vivo* experiments, rats were injected with saline, BMSCs expressing GFP (GFP‐BMSCs) or BMSCs expressing GFP‐P‐gp (MDR1‐GFP‐BMSCs). After dexamethasone induction, adipogenesis was determined by measuring PPAR‐γ expression and fatty marrow, whereas osteogenesis was detected by measuring Runx2 expression, trabecular parameters and the mineral apposition rate, followed by evaluation of the incidence of ONFH. Overexpression of P‐gp in BMSCs resulted in markedly decreased expression of adipogenic markers and increased expression of osteogenic markers. Compared with rats injected with saline, rats injected with GFP‐BMSCs showed reduced ONFH, and the injected GFP‐positive BMSCs attached to trabecular surfaces and exhibited an osteoblast‐like morphology. Compared with the rats injected with BMSCs expressing GFP alone, rats injected with BMSCs overexpressing GFP and P‐gp showed lower adipocytic variables, higher osteogenic variables and lower incidence of ONFH. Overexpression of P‐gp inhibited BMSC adipogenesis and promoted osteogenesis, which reduced the incidence of steroid‐induced ONFH.

## Introduction

Glucocorticoid use is the most common risk factor for non‐traumatic osteonecrosis of the femoral head (ONFH), a progressive and refractory disease with a high disability rate in young and active patients with an average age at onset of 38 years 1, 2, 3. Due to the young age of many of these patients, total hip replacements exhibit poor prosthetic durability. Although head‐preserving treatments, including core decompression, osteotomy, bone graft, and bone marrow and/or tantalum rod implantation, have been shown to be effective in the early stages of ONFH, the clinical results have not been consistently satisfactory 4, 5. Therefore, the precise mechanism of steroid‐induced ONFH should be further studied.

Recently, multidrug resistance gene 1 (MDR1, ABCB1) expression has been reported to be statistically related to the development of steroid‐induced ONFH 6, 7, 8, 9, 10. However, the precise mechanism remains unknown, which limits the application of this gene in strategies to prevent steroid‐induced ONFH. The MDR1 gene encodes for transmembrane P‐glycoprotein (P‐gp), which functions as an efflux transporter 6, 7, 8, 9, 10. Because glucocorticoid hormones are substrates of P‐gp, enhanced P‐gp activity can reduce the intracellular availability and accumulation of glucocorticoids by pumping these molecules out of cells 9, 10. In addition, because increased glucocorticoid levels in bone marrow‐derived multipotent stromal cells (BMSCs) promote adipogenesis 11, we hypothesized that enhanced P‐gp activity might inhibit BMSC adipogenesis by reducing glucocorticoid accumulation in BMSCs. Furthermore, because excessive BMSC adipogenesis has been regarded as the important mechanism of steroid‐induced ONFH 7, 10, 12, 13, 14, we also hypothesized that P‐gp might decrease the risk of ONFH by regulating adipogenic and osteogenic differentiation of BMSCs. To test our hypotheses and obtain information that can be used for the prevention of steroid‐induced ONFH, in this study, we investigated the mechanism by which P‐gp modulates BMSC differentiation. Bone marrow–derived multipotent stromal cells were isolated from rat bone marrow and transfected with lentiviral shuttle vectors carrying the MDR1 gene. We investigated the effects of P‐gp on steroid‐induced BMSC adipogenesis and osteogenesis *in vitro* as well as on the occurrence of steroid‐induced ONFH *via* the modulation of BMSC differentiation in an *in vivo* rat model.

## Materials and methods

### BMSC isolation, transfection and identification

Bone marrow–derived multipotent stromal cells were separated from the bone marrow of femurs of 6‐ to 7‐week‐old male Sprague–Dawley rats (provided by the Experimental Animal Centre of Tongji University, Shanghai, China) as previously described 15. Briefly, bone marrow cells were aseptically collected from the femurs with DMEM (Gibco, Gaithersburg, MD, USA), containing 10% foetal bovine serum (FBS; Biochemical Industries, Beth‐Haemek, Israel), then dispersed mechanically and suspended in PBS. The cells were carefully poured over a Percoll gradient (d = 1.073 g/ml; Sigma‐Aldrich, St. Louis, MO, USA) and centrifuged for 20 min. at 900 × g. The BMSC‐enriched intermediate zone was diluted with an equal volume of PBS and centrifuged at 900 × g for 10 min. The resulting pellet was suspended in complete DMEM supplemented with 10% FBS, 100 U/ml penicillin and 100 U/ml streptomycin. The cell suspension was plated at a density of approximately 5 × 10^8^ nucleated cells per millilitre in complete medium and cultured in 95% humidified air and 5% CO_2_ at 37°C. Non‐adherent hematopoietic cells were removed by changing the medium.

Third‐passage BMSCs were infected with lentiviral vectors carrying MDR1 and green fluorescent protein (GFP; Yusen Biotechnology, Shanghai, China) or a lentiviral vector carrying only GFP (Yusen Biotechnology). The transfected BMSCs were termed MDR1‐GFP‐BMSCs and GFP‐BMSCs, respectively. Bone marrow–derived multipotent stromal cells not subjected to transfection served as control cells. Twenty‐four hours after infection, the growth medium was replaced. When the BMSCs reached 50–70% confluence after an additional 48 hrs, the GFP‐positive BMSCs (GFP‐BMSCs or MDR1‐GFP‐BMSCs) were selected by flow cytometry to establish the stable transgenic cell lines. For flow cytometric analysis, BMSCs were suspended in PBS containing 10% FBS to a final density of 2 × 10^6^ cells/ml. A minimum of 10,000 events was collected for each analysis. Flow cytometry and cell sorting for GFP fluorescence were performed using a FACSCalibur flow cytometer (Becton Dickinson, San Jose, CA, USA). The resulting cells were resuspended in DMEM containing 10% FBS and allowed to reattach to culture dishes. For phenotypic characterization, approximately 2 × 10^5^ BMSCs from cell cultures were suspended in 1‐ml PBS supplemented with 10% FBS and then incubated with fluorescein isothiocyanate (FITC)‐conjugated CD34 (BD Bioscience Pharmingen, San Jose, CA, USA), phycoerythrin (PE)‐conjugated CD 44 (BD Bioscience, San Diego, CA, USA) or PE‐conjugated CD 29 (BD Bioscience Pharmingen) at a dilution rate of 1:100 for 40 min. at 4°C. After staining, the cell suspensions were washed three times and fixed in 2% paraformaldehyde. The flow cytometric analysis was carried out using a FACSCalibur flow cytometer with rat immunoglobulin G1‐FITC (BD Biosciences Pharmingen) as isotype controls. CellQuest software (Beckman Coulter, Brea, CA, USA) was employed for analysis.

### Expression and activity of P‐gp in BMSCs

According to a method previously described by Parasrampuria *et al*. 16, P‐gp activity was evaluated according to the rhodamine 123 efflux rate (RER). Rhodamine 123 (Sigma‐Aldrich) is a fluorescent dye that can be combined with and pumped out of cells by P‐gp. Cyclosporin A (Sigma‐Aldrich) was used to specifically prevent P‐gp–mediated rhodamine 123 efflux. Cell samples were digested with 0.05% trypsin‐0.02% ethylenediaminetetraacetic acid (EDTA; Gibco), and BMSCs were suspended in DMEM containing 10% FBS and 1 μg/ml rhodamine 123. The samples were maintained in a humidified atmosphere of 5% CO_2_ at 37°C for 45 min. to allow uptake, then centrifuged at 200 × g for 10 min., washed twice with PBS containing 10% FBS, resuspended with or without 3600 μg/l cyclosporin A in DMEM containing 10% FBS and incubated in a humidified atmosphere of 5% CO_2_ at 37°C for 30 min. The mean fluorescence intensity (MFI) of rhodamine 123 was immediately determined with a FacsAria flow cytometer (Becton Drive, Franklin Lakes, NJ, USA) as follows: RER = [(MFI with cyclosporin A − MFI without cyclosporin A)/MFI without cyclosporin A]*100%. The excitation and emission wavelengths for rhodamine 123 were 488 and 525 nm, respectively. Fifty‐microlitre samples without rhodamine 123 were examined by flow cytometry as a control.

P‐glycoprotein expression was investigated by Western blot analysis using a monoclonal anti‐P‐gp antibody (Abcam, Cambridge, UK). Bone marrow–derived multipotent stromal cells were scraped in lysis buffer containing Tris buffer (250 mM, pH 7.4), 150 mM NaCl and 1% Triton X‐100 supplemented with a 1:100 protease inhibitor and 1:100 phosphatase inhibitor mixture (Sigma‐Aldrich). After centrifugation at 10,000 × g for 10 min., the protein concentration in the supernatant was measured with a protein assay kit (Bio‐Rad, Mississauga, ON, Canada). Fifty micrograms of each preparation was loaded on 10% SDS polyacrylamide gels. After electrophoresis, the proteins were transferred onto a nitrocellulose membrane (Schleicher & Schuell, Dassel, Germany) and immunolabelled with monoclonal anti‐P‐gp antibody (diluted 1:20) overnight at 4°C. Horseradish peroxidase (HRP)‐labelled goat anti‐mouse (Santa Cruz Biotechnology, Santa Cruz, CA, USA) antibody was used as the secondary antibody. The protein was detected with enhanced chemiluminescence (Amersham Life Sciences, Arlington Heights, IL, USA), and the membranes were exposed to autoradiographic films for 20 min. (Eastman Kodak, Rochester, NY, USA). The relative expression was quantified by densitometry using the Quantity One software (Bio‐Rad Labs, Richmond, CA, USA) and calculated according to the reference bands of glyceraldehyde‐3‐phosphate dehydrogenase (GAPDH).

### Dexamethasone accumulation in BMSCs

Bone marrow–derived multipotent stromal cells were seeded at a density of 1 × 10^5^/ml in 12‐well plates for 3 days and then exposed to 1‐μM dexamethasone (DEX; Sigma‐Aldrich) for 24 hrs. The samples were digested with 0.05% trypsin‐0.02% EDTA (Gibco) and rinsed twice with PBS. Intracellular DEX levels were measured using a commercial competitive ELISA for DEX determination (Neogen Corporation, Lansing, MI, USA) after the cells were scraped in the lysis buffer described above (Sigma‐Aldrich), according to the manufacturer's instructions for the ELISA.

### BMSC differentiation

#### Adipogenesis

Bone marrow–derived multipotent stromal cells were seeded at a density of 1 × 10^5^/ml in 12‐well plates and cultured in basal medium (DMEM + 10% FBS). After 3 days, 1‐μM DEX was added to the basal medium for 14 days. The induced cells were re‐fed every 3 days with adipocyte maintenance medium (adipocyte differentiation medium without the IBMX and rosiglitazone). At day 14 of differentiation, the cells were fixed and stained with Oil Red O (Sigma‐Aldrich) to detect lipid droplets in the cytoplasm. For triglyceride (TG) level determination, the BMSCs from all groups were lysed with cell lysate (Sigma‐Aldrich) for 10 min. and centrifuged at 700 × g for 10 min. Triglyceride levels in the supernatants were determined with a fully automated Modular‐T Biochemical Analyzer (Roche Diagnostics, Mannheim, Germany).

#### Osteogenesis

Bone marrow–derived multipotent stromal cells were cultured in high‐glucose DMEM supplemented with 10% FBS, 50 mg/ml L‐ascorbate‐2‐phosphate, 1‐μM DEX and 10‐mM β‐glycerophosphate for 14 days. The cells were stained using an alkaline phosphatase (ALP) staining kit (Shanghai Hongqiao Chemical Reagent Inc., Shanghai, China), and ALP activity in the BMSCs was quantified with an ALP diagnostic kit (Abcam, Cambridge, MA, USA) according to the manufacturer's instructions.

#### Peroxisome proliferator‐activated receptor and runt‐related transcription factor 2 expression in induced BMSCs

The induced BMSCs were rinsed twice with PBS and scraped in the previously described lysis buffer (Sigma‐Aldrich). After centrifugation at 25,000 × g for 20 min., the protein content of the obtained extracts was determined with a protein assay kit (Bio‐Rad, Canada). For Western blot analysis, the proteins were immunolabelled with monoclonal antibodies directed against peroxisome proliferator‐activated receptor‐γ (PPAR‐γ) and runt‐related transcription factor 2 (Runx2; Santa Cruz Biotechnology). The method was similar to that used for the determination of P‐gp expression. Peroxisome proliferator‐activated receptor‐γ and Runx2 expression was measured by densitometry and normalized to that of GAPDH.

### Rat model for BMSC transplantation

The protocol for animal experiments was approved by the Committee of Animal Experimentation of Tongji University. One hundred and twenty male Sprague–Dawley rats weighing 250–300 g were raised in normal conditions of controlled temperature (24 ± 2°C) and humidity (55 ± 2%) at the experimental animal centre of Tongji University. The rats were divided into three groups (*n* = 40 per group) that received tail vein injections of 1‐ml normal saline (control group), 1 × 10^7^ GFP‐BMSCs (GFP‐BMSC transplant group) or 1 × 10^7^ MDR1‐GFP‐BMSCs (MDR1‐GFP‐BMSC transplant group). After 2 weeks, 20 rats from each group were anesthetized and killed to assess the transplantation efficiency of BMSCs. From the 15th day after injection, the remaining rats in all groups received weekly injections of 20 mg/kg DEX and were trained on a treadmill twice per week according to the method described by Ma *et al*. 17. After 10 additional weeks, all rats were anesthetized and killed. Bone labelling was achieved with an intraperitoneal injection of tetracycline (15 mg/kg, oxytetracycline; Pfizer, New York, NY, USA) 12 and 2 days before death. Ten left femoral heads from each group were harvested for Western blot analysis. An additional 10 left femoral heads from each group were harvested to determine the mineral apposition rates. The right proximal femurs and femoral heads were fixed in 2% paraformaldehyde for 48 hrs prior to micro‐computed tomography (micro‐CT) examination, after which they were prepared for paraffin embedding after decalcification with 10% EDTA solution. The femoral heads were cut along the coronal plane parallel to the longitudinal axis of the femur to prepare slices for the histological evaluation.

### Assessment of BMSC transplantation efficiency by flow cytometry

Two weeks after BMSCs were injected into rats, the same protocol described in section 2.1 was employed to isolate BMSCs from the femurs of the recipient rats. Upon resuspension in DMEM with 10% FBS, GFP‐expressing BMSCs were counted using a FACSCalibur flow cytometer (Becton Dickinson) to determine the transplantation efficiency. The GFP intensities were obtained by gating a two‐parameter forward‐ and sidescatter cytogram to a one‐parameter green fluorescence intensity plot. PBS was used as the negative control, and FITC conjugated to fluorescent CaliBRITE beads (Becton Dickinson) was used as the positive control as well as to calibrate the instrument. The percentage of GFP‐expressing transplanted cells was determined. Three replicates were performed for every flow cytometric experiment. The transplantation efficiency of BMSCs was quantified as the percentage of GFP‐positive BMSCs among the femoral BMSCs.

### P‐gp, PPAR‐γ and Runx2 expression in femoral heads

The left femoral heads were snap frozen in liquid nitrogen, crushed with a tissue grinder in RIPA (Radio Immunoprecipitation Assay) buffer and lysed at 4°C for 30 min. The lysate was subsequently centrifuged at 15,000 × g at 4°C for 10 min. Monoclonal anti‐P‐gp, anti‐PPAR‐γ and anti‐Runx2 antibodies (Santa Cruz Biotechnology) were used for immunolabelling, and Western blot analysis was performed as described above.

### Assessment of bone parameters by micro‐CT

Proximal sections of the dissected formalin‐fixed femurs were scanned using a SkyScan 1072 microtomograph (SkyScan, Antwerp, Belgium) at a voxel size of 11.89 μm. For calculation of trabecular bone parameters, a region of interest (ROI, 1.5 mm × 1.5 mm × 0.5 mm) containing only trabecular bone was selected in the femoral head. The following morphometric parameters were then calculated for the bone within the ROI: bone mineral density (BMD, g/cm^3^), trabecular bone volume fraction (bone volume divided by total volume, BV/TV), trabecular thickness (Tb.Th, μm) and trabecular number (Tb.N, 1/μm).

### BMSC adipogenesis and GFP immunostaining in the femoral head

Sections of the harvested femoral heads were stained with haematoxylin–eosin. Micrographs were captured for each slice of every femoral head sample under 400× magnification. Osteonecrosis was diagnosed based on the presence of empty lacunae or pyknotic nuclei of osteocytes in the bone trabeculae, accompanied by surrounding bone marrow cell necrosis 3, 17. The rate of empty lacunae in the bone was evaluated microscopically. During the examination of the trabeculae at 400× magnification, the rate of empty bone lacunae was calculated in each randomly chosen field. Image‐Pro Plus 6.0 software (Media Cybernetics, Silver Spring, MD, USA) was used to assess adipogenesis according to the adipose tissue area, number of adipocytes and adipocyte perimeter. The adipose tissue area was expressed as the percentage of the total bone marrow area occupied by adipocytes. The number of adipocytes was expressed as the total number of adipocytes per bone marrow area.

Because GFP fluorescence was difficult to distinguish from the tetracycline fluorescence, GFP expression in the sections was determined by immunostaining with an avidin–biotin–peroxidase procedure. After deparaffinization and rehydration, the sections were treated with 0.5% hydrogen peroxide to block endogenous peroxidase activity, incubated with normal rabbit serum (DAKO, Hamburg, Germany) for 30 min. at 37°C, and incubated with polyclonal rabbit anti‐GFP antibody (diluted 1:20; Abcam) overnight in a moist chamber at 4°C. On the following day, the tissue sections were incubated with secondary goat anti‐rabbit antibody (DAKO) and HRP‐labelled streptavidin (DAKO). The final reaction product was revealed by exposure to 0.03% diaminobenzidine (DAB; Sigma‐Aldrich), and the nuclei were counterstained with haematoxylin. For each specimen, a negative control was obtained by staining a sample with only secondary antibody.

### Mineral apposition rate

The harvested left femurs were fixed in 40% ethanol for 24 hrs and subsequently maintained in 70% ethanol until processing. The samples were dehydrated in graded concentrations of ethanol and embedded undecalcified in methylmethacrylate (Sigma‐Aldrich). Longitudinal frontal sections of the proximal femur were cut at a thickness of 7 μm using a Polycut S heavy‐duty microtome (Leica Instruments, Heidelberg, Germany). The mineral apposition rate (MAR, μm/day) in these sections was measured by double tetracycline labelling using Image‐Pro Plus 6.0 software.

### Statistical analysis

All data are expressed as the mean ± S.D. All analyses were performed using Stata 7.0 software (StataCorp, College Station, TX, USA). The Bonferroni correction of anova and Kruskal–Wallis test results were used to detect significant differences among groups. The incidence of ONFH was analysed by the Pearson chi‐square test. *P* < 0.05 was considered statistically significant.

## Results

### MDR1 overexpression in BMSCs promoted P‐gp activity and expression *in vitro*

**Figure 1:**
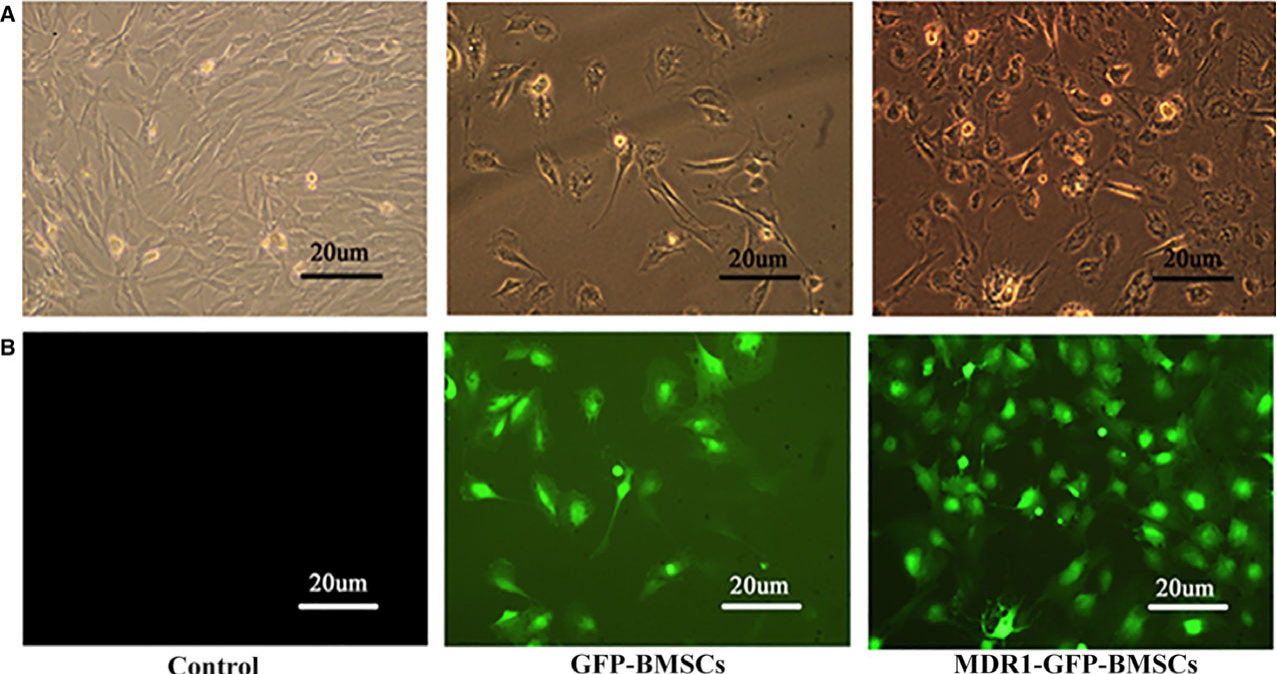
Stability of transgenic BMSCs. (**A**) Inverted phase contrast microscopy images showing morphology of control BMSCs, GFP‐BMSCs and MDR1‐GFP‐BMSCs. (**B**) Fluorescence microscopy images of control BMSCs, GFP‐BMSCs and MDR1‐GFP‐BMSCs.

Upon plating, the majority of the attached bone marrow–derived cells exhibited a fibroblast‐like spindle shape and began to proliferate. After 7 days, these fibroblast‐like cells had reached confluence and formed a tightly packed monolayer. Using flow cytometry, we confirmed that the cells showed a surface antigen expression pattern characteristic of BMSCs (CD34^−^, CD44^+^ and CD29^+^). After the BMSCs were infected with lentiviral vectors carrying GFP and the MDR1 gene or only GFP, the GFP‐positive BMSCs were selected to establish stable transgenic GFP‐BMSCs and MDR1‐GFP‐BMSCs (Fig. 1).

**Figure 2:**
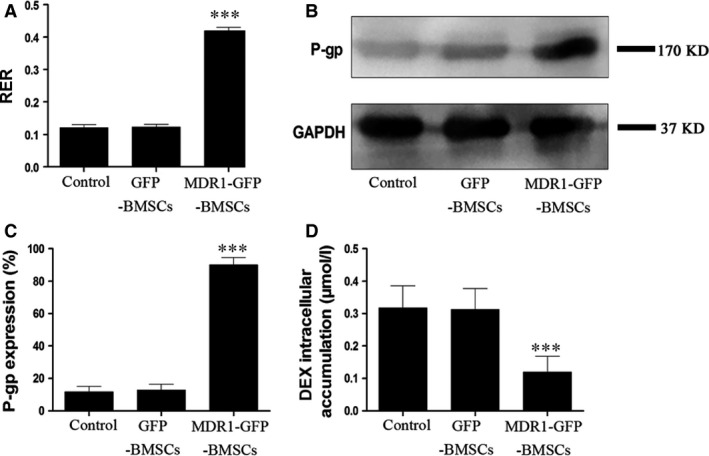
P‐gp activity and expression in control BMSCs, GFP‐BMSCs and MDR1‐GFP‐BMSCs. (**A**) RER. (**B**) Representative images of Western blots for P‐gp and GAPDH expression. (**C**) Quantitative results for relative P‐gp expression. (**D**) Intracellular DEX accumulation. Data are expressed as mean ± S.D. ***P < 0.01 relative to control BMSCs and GFP‐BMSCs.

The RER of MDR1‐GFP‐BMSCs was the highest among the three cell groups (Fig. 2A), indicating that P‐gp activity was highest in these cells among all groups. No significant difference was detected between the RERs for control BMSCs and GFP‐BMSCs.

P‐glycoprotein expression in the different BMSC groups was assessed by Western blotting, and P‐gp expression in MDR1‐GFP‐BMSCs was the highest among the three groups (Fig. 2B and C). No difference in P‐gp expression was observed between control BMSCs and GFP‐BMSCs.

### Effects of P‐gp overexpression on BMSC differentiation potential *in vitro*

Dexamethasone accumulation in MDR1‐GFP‐BMSCs was the lowest among the groups, and no significant difference in DEX accumulation was observed between control BMSCs and GFP‐BMSCs (Fig. 2D).

**Figure 3:**
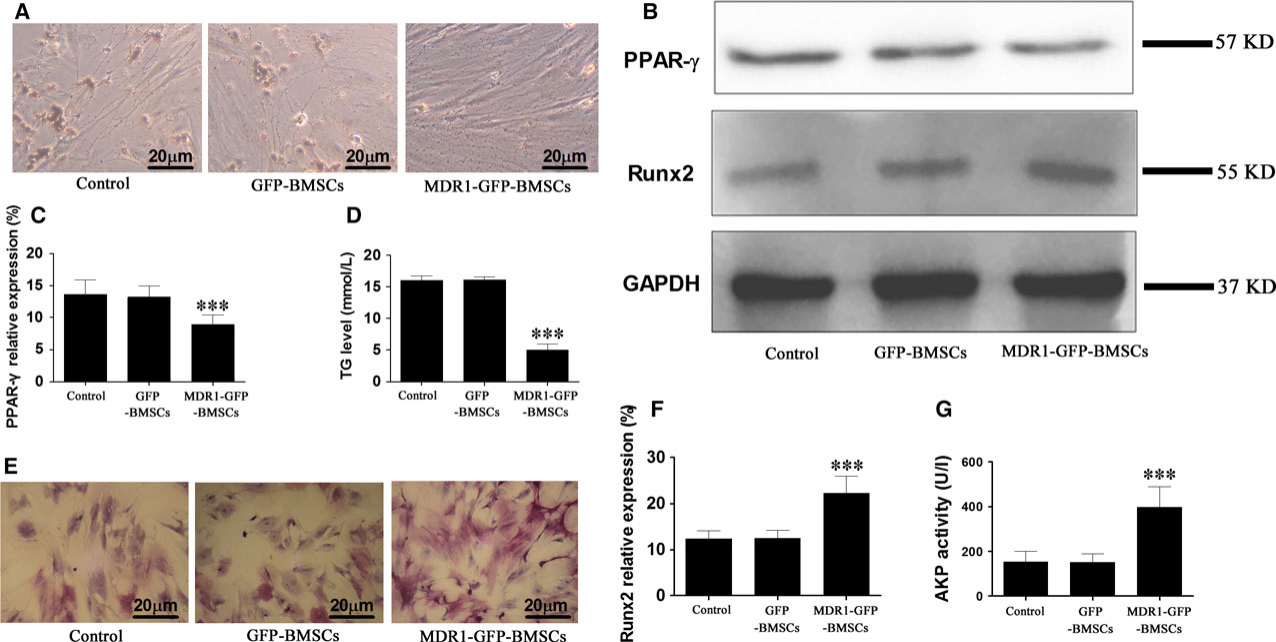
BMSC adipogenesis and osteogenesis *in vitro*. (**A**) Oil Red O staining of BMSCs on day 14 indicating adipogenesis. (**B**) Representative Western blots for PPAR‐γ and Runx2 expression. (**C**) Densitometric quantification of PPAR‐γ protein expression relative to GAPDH expression. (**D**) TG levels in BMSCs. (**E**) ALP staining of BMSCs on day 14 indicating osteogenesis. (**F**) Densitometric quantification of Runx2 protein expression relative to GAPDH expression. (**G**) Evaluation of ALP activity in BMSCs. Data are expressed as mean ± S.D. ***P < 0.01 relative to control BMSC and GFP‐BMSCs.

Upon induction of adipogenesis, BMSCs changed from spindle‐shaped to round‐shaped. Similar adipogenesis was observed between control BMSCs and GFP‐BMSCs, whereas fewer MDR1‐GFP‐BMSCs differentiated into adipocytes (Fig. 3A). Moreover, PPAR‐γ expression and TG accumulation of MDR1‐GFP‐BMSCs were lower than those in control BMSCs and GFP‐BMSCs, and no significant differences in PPAR‐γ expression or TG level were observed between control BMSCs and GFP‐BMSCs (Fig. 3B–D).

Upon induction of osteogenesis, ALP staining in MDR1‐GFP‐BMSCs was the strongest among all groups (Fig. 3E), and no obvious difference in ALP staining was observed between control BMSCs and GFP‐BMSCs (Fig. 3E). Furthermore, Runx2 expression and ALP activity in MDR1‐GFP‐BMSCs were the highest among the groups, and no significant differences in Runx2 expression or ALP activity were observed between control BMSCs and GFP‐BMSCs (Fig. 3B, F and G).

### Effects of MDR1‐expressing BMSC transplantation on femoral bone *in vivo*

**Figure 4:**
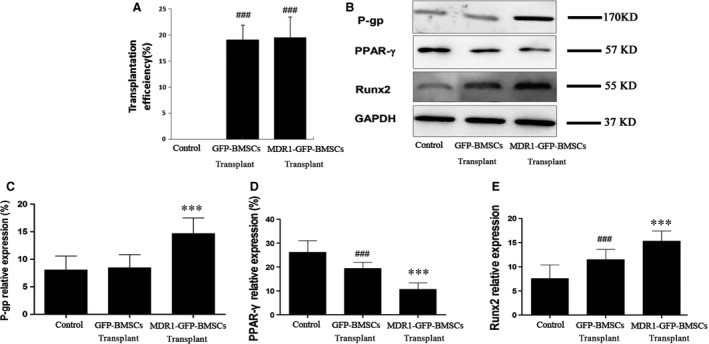
Transplantation efficiency of BMSCs in femurs and quantification of relative P‐gp, PPAR‐γ and Runx2 protein expression in the femoral head. (**A**) Transplantation efficiency of BMSCs was quantified as the percentage of GFP‐positive BMSCs among the femoral bone marrow cells as measured by flow cytometry. Data are expressed as mean ± S.D. (**B**) Representative images of Western blots. Densitometric quantification of (**C**) P‐gp, (**D**) PPAR‐γ and (**E**) Runx2 protein expression relative to GAPDH expression. Data are expressed as mean ± S.D. ***P < 0.01 relative to control group and GFP‐BMSC transplant group. ^###^ *P* < 0.01 relative to control group.

Flow cytometric analysis revealed the number of GFP‐positive cells in the bone marrow of rats that received MDR1‐GFP‐BMSCs was approximately equal to that in the bone marrow of rats that received GFP‐BMSCs, and as expected, no GFP‐positive cells were found in the bone marrow of rats that received saline (Fig. 4A).

According to Western blot analyses, P‐gp expression in the femoral head was highest in rats that received MDR1‐GFP‐BMSCs, and no significant difference in P‐gp expression was observed in the femoral heads of rats that received saline injection or GFP‐BMSCs (Fig. 4B and C). Peroxisome proliferator‐activated receptor‐γ expression in the femoral head of rats that received MDR1‐GFP‐BMSCs was the lowest among the groups and was lower in rats that received GFP‐BMSCs than in those that received saline (Fig. 4B and D). In contrast, Runx2 expression in the femoral head of rats that received MDR1‐GFP‐BMSCs was the highest among the groups and was lower in rats that received GFP‐BMSCs than in those that received saline (Fig. 4B and E).

**Figure 5:**
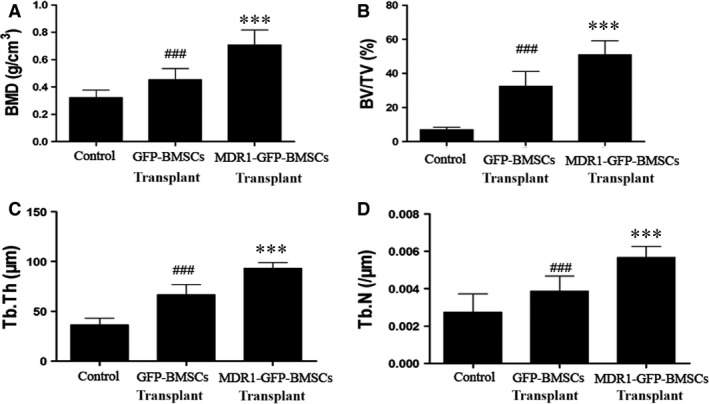
Determination of bone histomorphometric parameters in femoral head. (**A**) BMD, (**B**) BV/TV, (**C**) Tb.Th and (**D**) Tb.N. Data are expressed as mean ± S.D. ***P < 0.01 relative to control group and GFP‐BMSC transplant group. ^###^ *P* < 0.01 relative to control group.

The BMD, BV/TV, Tb.Th and Tb.N values for the femoral head were higher in rats that received MDR1‐GFP‐BMSCs than in the other groups and were higher in rats that received GFP‐BMSCs than in rats injected with saline (Fig. 5A–D).

### Differentiation of transplanted BMSCs *in vivo* and the effect on the incidence of ONFH

**Figure 6:**
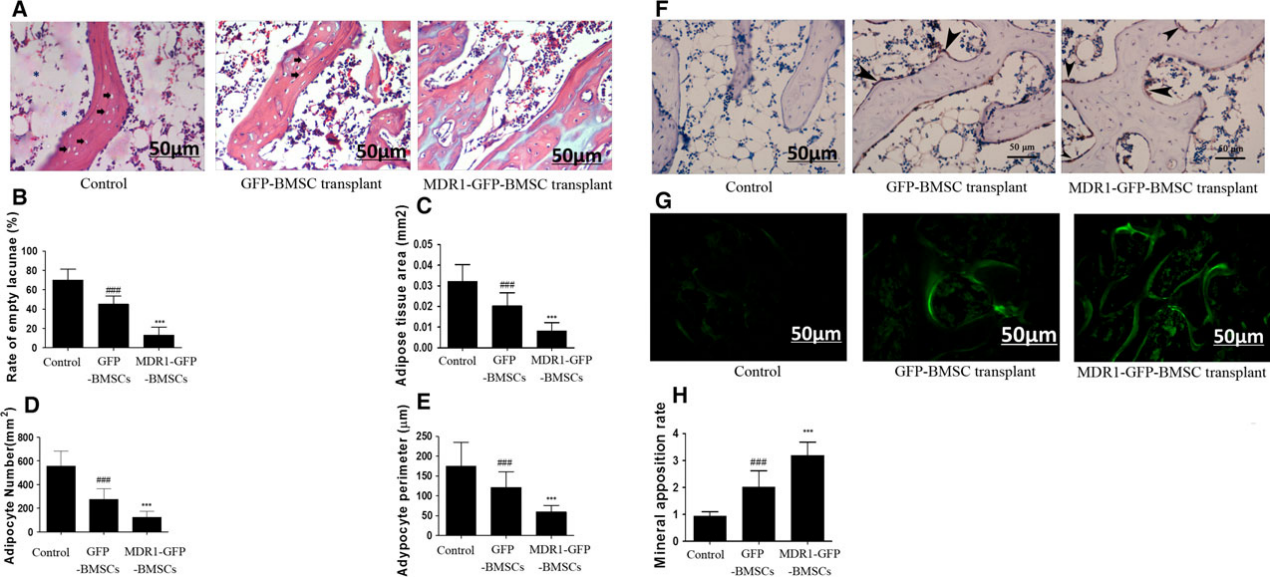
Analyses of adipogenesis and osteogenesis in the femoral head. (**A**) Haematoxylin–eosin staining for observation of empty lacunae (*black arrow*), thickness and density of trabeculae, bone marrow cell necrosis (*), and numbers and sizes of adipocytes in the femoral head of rats that received saline (control treatment), GFP‐BMSCs or MDR1‐GFP‐BMSCs. (**B**) Rate of empty lacunae. (**C**) Adipose tissue area. (**D**) Adipocyte number. (**E**) Adipocyte perimeter. (**F**) Immunohistochemical staining for GFP in sections of the femoral head. Positive staining mainly occurred in cells (*black arrowhead*) attached to the surface of the trabeculae and shaped like osteoblasts after transplantation of GFP‐BMSCs or MDR1‐GFP‐BMSCs. (**G**) Tetracycline fluorescence indicating new bone formation. (**H**) MAR. In all panels, data are expressed as mean ± S.D. ***P < 0.01 relative to control group and GFP‐BMSC transplant group. ^###^ *P* < 0.01 relative to control group.

In rats that received MDR1‐GFP‐BMSCs, the extent of adipogenesis in the femoral heads was the lowest among the groups, and the trabeculae were densest and thickest in the femoral heads of this group. In rats that received GFP‐BMSCs, adipogenesis in the femoral head was less extensive than in rats that received saline. The trabeculae after GFP‐BMSC transplantation were denser and thicker than those after saline injection, but sparser and slimmer than those formed after MDR1‐GFP‐BMSC transplantation (Fig. 6A).

The adipocytic parameters, including adipose tissue area, adipocyte number and adipocyte perimeter, in the femoral head of rats that received MDR1‐GFP‐BMSCs were the lowest among the groups, and in rats that received GFP‐BMSCs, these parameters were lower than in rats injected with saline (Fig. 6C–E).

The incidence of ONFH (10%, *P* < 0.01) and the rate of empty lacunae (13.15 ± 8.27, *P* < 0.01) were the lowest in rats that received MDR1‐GFP‐BMSCs among all groups and were lower in rats that received GFP‐BMSCs (55%; *P* < 0.05, 45.30 ± 8.25%; *P* < 0.01) than in the control group (90%, 70.05 ± 11.30%; Fig. 6B).

Moreover, immunostaining analyses revealed GFP expression in the femoral heads of rats that received GFP‐BMSCs or MDR1‐GFP‐BMSCs, and the GFP‐positive cells were shaped like osteoblasts and attached to trabecular surfaces (Fig. 6F). As an indicator of osteogenesis, the tetracycline fluorescence band was the widest in the femoral head of rats that received MDR1‐GFP‐BMSCs and greater in the group that received GFP‐BMSCs than in the control group (Fig. 6G). In addition, the MAR in the femoral head of rats that received MDR1‐GFP‐BMSCs was the highest among the groups (Fig. 6H).

## Discussion

As many as 9–40% of patients receiving long‐term glucocorticoid therapy develop glucocorticoid‐induced ONFH, the mechanisms of its pathogenesis are not well understood. Moreover, the physiological roles of BMSCs in bone regeneration are ill‐defined. In this study, BMSCs transfected with the MDR1 gene exhibited increased P‐gp expression and reduced intracellular DEX accumulation, which inhibited adipogenesis and promoted osteogenesis *in vitro*. Injection of MDR1‐GFP‐BMSCs in rats resulted in enhanced osteogenic changes in the femoral head, whereas adipocytic changes were inhibited, and the incidence of steroid‐induced ONFH was reduced. Together, these observations suggest that P‐gp decreases the risk of steroid‐induced ONFH by modulating BMSC adipogenesis and osteogenesis.

Bone marrow–derived multipotent stromal cells offer excellent proliferative capability and versatility for pluripotent differentiation into osteoblasts, adipocytes, chondrocytes, vascular endothelial cells and nerve cells under different induction conditions 18, 19, 20, 21, 22. Exogenous synthetic glucocorticoids, such as DEX, have been shown to be key regulators of adipogenesis and osteogenesis 22. For example, Ghali *et al*. were able to simultaneously induce BMSC adipogenesis and osteogenesis using the same concentration of DEX 21. However, BMSC adipogenesis and osteogenesis are competitively balanced, and for this reason, osteogenesis of BMSCs is inhibited when adipogenesis is enhanced 2, 14, 23. Our findings in rats that received MDR1‐GFP‐BMSCs confirm the reciprocal balance that enhanced osteogenesis must be accompanied by inhibited adipogenesis. By comparing BMSC differentiation in the femoral head of rats that received MDR1‐GFP‐BMSCs to that in rats that received either GFP‐BMSCs or saline, we found that the inhibited adipogenesis and enhanced osteogenesis of BMSCs can likely be attributed to P‐gp overexpression.

P‐glycoprotein is a transmembrane glycoprotein measuring 170 kD and encoded by the 4.5‐kb MDR1 gene, which is located on human chromosome 7q21.12 6, 7, 8, 9, 10. In this study, we used a lentiviral vector carrying the human MDR1 gene to transfect rat BMSCs and succeeded in increasing P‐gp activity and expression in the transfected BMSCs. Importantly, MDR1‐GFP‐BMSCs exhibited the same proliferative and differentiation capabilities as the control BMSCs, which showed that P‐gp overexpression had no direct effect on BMSC differentiation without DEX. Glucocorticoids are substrates of P‐gp and can be pumped out of cells 9, 10. Our findings further revealed that DEX accumulation was reduced in P‐gp overexpressing BMSCs. According to recent reports 10, 11, 20, 21, 23, 24, 25, 26, the DEX concentration plays a key role in BMSC adipogenesis and more adipocytic cells are derived from BMSCs with an increasing concentration of Dex. Moreover, lower DEX concentrations decrease PPAR‐γ expression and inhibit BMSC adipogenesis 2, 11, 25, 26. In this study, P‐gp overexpression decreased PPAR‐γ expression and inhibited BMSC adipogenesis with a concomitant decrease in the intracellular DEX level. Because PPAR‐γ is the key regulator of BMSC adipogenesis 18, 27, 28, we conclude that P‐gp inhibited PPAR‐γ‐mediated BMSC adipogenesis by pumping intracellular DEX out of the cells.

Runt‐related transcription factor 2, a transcription factor, is essential for osteoblastic differentiation, bone formation and maintenance 29. In the early stage of BMSC osteogenesis, ALP activity increases along with Runx2 expression 30. In this study, Runx2 expression and ALP activity increased with P‐gp overexpression, suggesting that P‐gp enhanced DEX‐induced BMSC osteogenesis. It has been reported that PPAR‐γ can inhibit the expression of Runx2, thereby inhibiting osteogenesis 23. Sun *et al*. showed that Runx2 expression increases and promotes BMSC osteogenesis when PPAR‐γ expression is downregulated by microRNAs 31. In this study, P‐gp overexpression inhibited PPAR‐γ expression by decreasing the intracellular concentration of DEX. Therefore, we concluded that P‐gp enhanced BMSC osteogenesis by inhibiting PPAR‐γ expression.

Studies have shown that steroid‐induced ONFH may be a result of abnormal differentiation of BMSCs 32, 33. To study the effects of P‐gp on steroid‐induced ONFH, we transplanted the same number of GFP‐BMSCs or MDR1‐GFP‐BMSCs into rats *via* the tail vein. According to the study by Cui *et al*. 34, BMSCs transplanted *via* tail vein injection can be incorporated into the femoral bone marrow. Our results also showed the transplanted GFP‐expressing BMSCs resided in the femoral bone marrow and femoral head. In this study, with GFP expression detected in the femoral heads of rats that received GFP‐BMSCs and those that received MDR1‐GFP‐BMSCs, and with P‐gp expression in the femoral head being greater in rats that received MDR1‐GFP‐BMSCs, we conclude that the transplanted BMSCs had been transferred into the femoral heads of the rats, which resulted in an increase in the mesenchymal stem cell pool in the femoral head. These observations are in line with those of Hernigou *et al*. 12, 13, who confirmed that a decrease in the pool of mesenchymal stem cells in the proximal femur is responsible for steroid‐induced ONFH. In this study, the incidence of ONFH in rats that received GFP‐BMSCs or MDR1‐GFP‐BMSCs and thus had an increased pool of BMSCs was lower than that in the control group. In addition, the increased pool of BMSCs improved the trabecular parameters and new bone formation in the femoral heads. According to our GFP immunostaining results, many GFP‐positive cells shaped like osteoblasts and attached to the surface of trabecula were observed after transplantation of GFP‐BMSCs or MDR1‐GFP‐BMSCs, and these processes were associated with increased expression of a marker of BMSC osteogenesis (Runx2). Therefore, we speculated that transplanted BMSCs may differentiate into osteoblasts to improve bone formation and prevent ONFH by repairing the damage induced by glucocorticoids. However, we noticed that the incidence of ONFH in rats that received GFP‐BMSCs or MDR1‐GFP‐BMSCs differed, although these rats both had similarly increased mesenchymal stem cell pools, based on the transplantion of the same number of BMSCs *via* the tail vein. We also found that the high expression and activity of P‐gp was related to the lower incidence of ONFH. Based on the results of our *in vitro* study, we inferred that P‐gp overexpression inhibited adipogenesis and stimulated osteogenesis in BMSCs to decrease the incidence of ONFH.

In addition to BMSCs, P‐gp is also expressed in osteoblasts, chondrocytes and vascular endothelial cells 10, 35, and the mechanisms of glucocorticoid‐induced ONFH are multifactorial, including apoptosis, vascular thrombosis, oxidative stress, *etc*. 4, 5. Therefore, the protective effects of P‐gp on other cell types in ONFH still require further elucidation. However, the ability of local modulation of P‐gp to inhibit ONFH has been well established, which can be used to develop a bone‐targeted drug to modulate P‐gp expression and activity in the skeleton to avoid osteonecrosis.

In conclusion, P‐gp overexpression in the femoral head contributed to the prevention of steroid‐induced ONFH. Although the mechanism of steroid‐induced ONFH is multifactorial, our results demonstrate that enhanced P‐gp activity and expression can inhibit adipogenesis and enhance osteogenesis of BMSCs *in vitro* and *in vivo*. These effects inhibited the development of fatty marrow and improved bone formation in the femoral head, which decreased the risk of steroid‐induced ONFH.

## Conflict of interest

Ning Han, Zengchun Li, Zhengdong Cai, Zuoqin Yan, Yingqi Hua and Chong Xu declare that they have no conflicts of interest.

## Author contribution

Ning Han contributed to the interpretation of data and to writing of the article; Zengchun Li contributed to the revision of the article; Zhengdong cai and Zuoqin Yan contributed to the research design and critical revision of the article; Yingqi Hua and Chong Xu contributed to the data collection.
